# Phase-matched electron–photon interactions enabled by 3D-printed helical waveguides

**DOI:** 10.1515/nanoph-2025-0297

**Published:** 2025-09-10

**Authors:** Masoud Taleb, Mohsen Samadi, Nahid Talebi

**Affiliations:** Institute of Experimental and Applied Physics, Kiel University, 24098 Kiel, Germany; Department of Electrical and Information Engineering, Kiel University, 24143 Kiel, Germany

**Keywords:** electron-driven photon source, cathodoluminescence, 3D printer, helical waveguide

## Abstract

The Smith–Purcell effect enables electromagnetic radiation across arbitrary spectral ranges by phase-matching the diffraction orders of an optical grating with the near-field of a moving electron. In this work, we introduce a novel approach using a helically shaped waveguide, where phase-matching is achieved through guided light within a helical optical fiber fabricated via two-photon polymerization using a 3D printer. Our results demonstrate that radiation from these structures precisely satisfies the phase-matching condition and is emitted directionally at specific angles, contrasting with the broad angular distribution characteristic of the traditional Smith–Purcell effect. Helical electron-driven photon sources establish a new paradigm, enabling 3D-printed structures to control electron-beam-induced radiation and, inversely, to facilitate light-induced efficient electron beam shaping and acceleration.

## 1 Introduction

Electron-driven photon sources are electromagnetic radiation sources integrated into electron microscopes as internal emitters [1], [2], [3], [4], with applications in spectral interferometry [5], [6] and time-resolved spectroscopy [7]. The main advantage of these sources is that they allow for generation of optical fields that are mutually coherent with respect to the evanescent near-field of the moving free-electron in vacuum and can be used for Ramsey-type interferometry of quantum objects in two-dimensional materials [8]. To realize such experiments, it is essential that the emitted light propagates collinearly with the electron trajectory, thereby enabling simultaneous excitation of the sample by both the generated light from the electron-driven photon source and the electron beam.

These sources typically consist of nanostructured metallic planar films that in interaction with electron beams generate light with specific radiation profiles, such as focused radiation [1] or vortex beams [8], [9]. While they provide a versatile platform for generating shaped electromagnetic radiation at specific photon energies defined by surface plasmon resonances, their relatively weak radiation intensities limit their effectiveness in nonlinear optics and pump-probe spectroscopy applications.

Hence, other radiation mechanisms from electron beams could be utilized to enhance the radiation intensity, particularly through phase-matching (synchronization) between the near-field of the moving electron and the induced polarizations in the material [11], [12], [13], [14], [15], [16]. Cherenkov radiation [16] and Smith–Purcell effect [17] are among radiation mechanisms enabled by phase-matching.

Cherenkov radiation is the radiation caused by electron beams travelling in a medium where the phase velocity of light is larger than the electron group velocity. Cherenkov radiation forms a light cone along the electron trajectory, with the cone angle *θ* specified by the phase-matching condition $nk_{0}\cos\theta =\omega \;v_{e}^{-1}$ , resulting in $\cos\theta =c{\left(nv_{e}\right)}^{-1}$ [3], [18]. Here, *k*_0_ is the radiation wavenumber, *ω* is its angular frequency, and *v*_*e*_ is the electron’s group velocity. This criterion however, neglects the fact that electron beams cannot propagate for long distances in materials, due to the strong elastic and inelastic scatterings from atoms and material excitations. Instead, electron beams travelling in vacuum near a vacuum/material interface and parallel to it provide an alternative [19], [20]. When the Cherenkov radiation is coupled to a waveguide mode copropagating with the electron, it leads to a strong, resonant, and phase-matched electron-photon interaction [21], [22]. However, the Cherenkov radiation in this case remains in the waveguide, making it inaccessible for far-field detectors.

Smith–Purcell effect mediates electron–photon interactions via coupling to optical grating modes, with a discrete set of wave numbers as *k*_*m*_ = *k*_0_ cos*θ* + 2*mπ*Λ^−1^, associated with optical rays propagating in various directions in vacuum [23], [24], [25]. Here, Λ is the grating period and *m* is an integer, defining the diffraction mode. The phase-matching scenario in this case is recast as $k_{m}=\omega \;v_{e}^{-1}$ . Generating coherent Smith–Purcell radiation with a tailored beam profile – such as a Gaussian beam aligned with the electron propagation direction – requires the use of cylindrical gratings or curved metamaterial structures, which present significant challenges for current nanofabrication technologies [26], [27].

Here, we demonstrate that phase-matching between a moving electron and the generated optical waves can be achieved using a waveguide shaped into a helical configuration. This geometry effectively extends the optical path length within the waveguide, thereby facilitating sustained phase-matching over longer interaction distances. Furthermore, the emitted light remains collinear with the electron trajectory and preserves a circular polarization, which is an important advantage for performing phase-locked photon-electron spectroscopy on two-dimensional materials, such as probing chiral excitons in transition metal dichalcogenides [28].

## 2 Results and discussions

**Figure 1:**
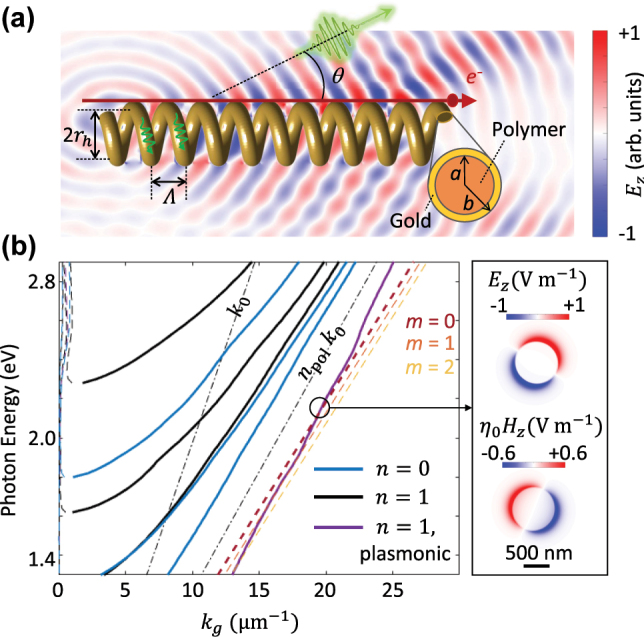
Phase-matched generation of Smith–Purcell radiation from a helical waveguide. (a) Topology of the structure, where a moving electron travels parallel with the screw axis of a helix. The helix is composed of a polymer fiber with a radius of *a* = 400 nm and with a 40 nm gold layer deposited on it. (b) Left: Calculated dispersion of the fiber modes. The dashed-dotted lines indicate the light lines in vacuum and fiber. Thin dashed lines show the attenuation constant and thick solid lines the phase constant. The red, orange, and yellow dashed lines specify the phase matching condition corresponding to a moving electron at the kinetic energy of 18 keV (Eq. (1) in the text). Right: Spatial profiles of the *z*-component of the electric and magnetic fields at the depicted point.

A 3D-printed helical waveguide is proposed and explored here (Figure 1(a)), that allows for the phase-matched generation of coherent light in interaction with a moving electron. The waveguide is composed of a polymer resin with the radius of 400 nm, where a thin layer of metal with the thickness of approximately 40 nm is deposited on it. The helix is fabricated using two-photon polymerization [29], [30], [31], [32], [33], [34] in a Nanoscribe Quantum X system with a 63x NA1.4 objective and an IP-Dip 2 resin with the refractive index of *n*_pol_ = 1.55. The radius of the helix, measured as the distance between the center of the waveguide to helix axis, is *r*_*h*_ = 1.76 μm, and the helical pitch is Λ = 5.85 μm.

For simplicity, we assume that the waveguiding modes remain unchanged when the straight fiber is transformed into a helix. The waveguide dispersion can be solved analytically (Figure 1(b); see Supplementary Note 1 for the solutions [35]). The modes of waveguides with cylindrical symmetries are generally decomposed versus the azimuthal order *n*, ranging from *n* = 0 (no azimuthal dependence; shown by blue lines in Figure 1(b)) to higher order modes with *n* ≥ 1 (shown by black and purple lines). The latter modes are hybrid in nature, i.e., they are in the form of the superposition of TE_*z*_ (transverse electric to *z*) and TM_*z*_ (transverse magnetic to *z*) modes. The fundamental mode of the waveguide is also a hybrid mode with *n* = 1 (purple line in Figure 1(b)), lying outside the light line in polymer. This mode has a plasmonic nature, with its field being tightly confined to the thin metallic region (Figure 1(b), right).

A moving electron propagating parallel to the helix axis at a distance of *r*_*h*_ + *b* + 20 nm, therefore not travelling through the matter, can excite the propagating modes of the waveguide. The sequential interaction of electrons with the waveguide at each turning point of the helix leads to the sequential emission of photons into guided modes. The optical path taken by the photons travelling within the waveguide between two turning points of the helix is $\sqrt{{\left(2\pi r_{h}\right)}^{2}+\Lambda^{2}}$ , and $k_{g}\sqrt{{\left(2\pi r_{h}\right)}^{2}+\Lambda^{2}}$ is the corresponding optical phase experienced by the traveling photons.

Within the same time interval, the electron travels the distance Λ and the incident field corresponding to the near-field of the moving electron undergoes a phase shift equal to Λ*ω*/*v*_*e*_. In the case of the constructive interaction between the emitted photons at both interaction points, the phase-matching condition leads to $k_{g}\sqrt{{\left(2\pi r_{h}\right)}^{2}+\Lambda^{2}}+2m\pi =\Lambda \omega /v_{e}$ . This condition is expressed as

(1) $$k_{g}=\left(\Lambda \omega /v_{e}-2m\pi \right)\cdot {\left({\left(2\pi r_{h}\right)}^{2}+\Lambda^{2}\right)}^{-\frac{1}{2}}.$$

The electron-induced guided waves within the waveguide gradually contribute to the radiation due to the sharp bending geometry of the helical structure. Equation (1) is a generalized Smith–Purcell radiation criterion, where the emitted photon paths are modified by accounting for traveling waves inside the waveguide. This generalized phase-matching condition reproduces the Smith–Purcell condition when neglecting the optical path taken by the photons travelling within the waveguide, by inserting *r*_*h*_ = 0 and inserting *k*_*g*_ = *k*_0_ cos*θ*, where *θ* is the angle between the electron trajectory and radiated optical rays. Similarly, it reproduces the Cherenkov condition by inserting *m* = 0 and *r*_*h*_ = 0.

The right side of Eq. (1) is provided as red, orange and yellow dashed lines in Figure 1(b), for an electron at the kinetic energy of 18 keV, and with *m* = 0, 1, and 2, respectively. Electrons having a kinetic energy (*U*_el_) in the range of 16 keV ≤ *U*_el_ ≤ 20 keV are able to couple to the plasmonic mode in a phase-matched condition with *m* = 0, whereas coupling to this mode for higher electron energies is only possible by higher diffraction orders, namely *m* > 0. The metallic layer serves a dual purpose in our configuration: it facilitates the coherent excitation of plasmonic waves and their interaction with electron beams, while also preventing charging of the helix under electron beam irradiation. This merged Cherenkov and Smith–Purcell emission mechanism significantly enhances the radiation. The emitted photons constitute a rate of approximately 0.02 photon per given electron (for a Helix with 11 turns), significantly higher than the emission from planar, plasmonic-based electron-driven photon sources emitting at the rate of approximately 10^−4^ per given electron, as confirmed by our measurements presented below. The photon statistics from our EDPHS structures have been obtained by utilizing a photon-counting detector (PCD) connected to our CL setup and calibrating the spectrograph counts by comparing the number of photons obtained via the PCD with the counts registered by the CCD camera, integrated over the spectral range of the EDPHS radiation. The acquisition time was set to either 200 ms or 250 ms, the detector gain was fixed at 1, the binning was 2, and the spectral acquisition was performed with a pixel size ranging from 10 nm to 20 nm.

Notably, in the case of pure Smith–Purcell emission – where the excitation of guided modes is neglected – the emission follows the classical Smith–Purcell condition, as previously discussed. For a 20 keV electron beam to generate light that propagates collinearly with the electron trajectory, the grating pitch must be approximately 224 nm. However, achieving such fine feature sizes exceeds the resolution limits of current 3D fabrication methods based on two-photon polymerization.

**Figure 2:**
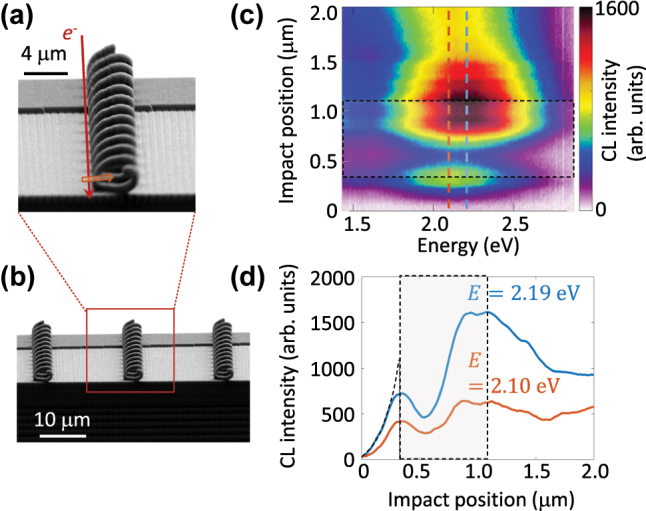
3D-printed helical waveguide. (a) Secondary electron image of a single 3D-printed helical waveguide. (b) SE image of a series of such waveguides arranged on a plateau. (c) CL spectra as a function of the electron impact position along the path indicated by the arrow in (a). The region within the core of the helical waveguide and its metallic shell is highlighted with a dashed, shaded box. (d) Linear plots of the CL intensity versus electron impact position at the specified photon energies.

The helical waveguides are positioned on top of a plateau fabricated at the same size of the helices (Figure 2(a) and (b) and Supplementary Figure S1). The structures are held by a special holder fabricated for precise alignment with respect to the electron beam, therefore the electron travels parallel with the helix axis (see Supplementary Figure S1). For measuring the radiation from electron beams interacting with the helical waveguide, a DELMIC SPARC cathodoluminescence (CL) spectroscopy and angle-resolve mapping system installed in a ZEISS SIGMA scanning electron microscope is used (Figure S1). A parabolic mirror positioned either above or below the helix gathers the radiation with the collection angular range of 1.46 π*sr*, and collimates it toward the analyzing path [36]. For the measurements shown below an electron beam at the kinetic energy of 18 keV and the current of 10 nA is used and the mirror is positioned below the helix.

Figure 2(c) shows the CL spectrum as a function of the electron impact position, varying from 320 nm away from the waveguide rim toward the helical axis until a distance of 500 nm from the axis. The intensity reaches a maximum at the photon energy of 2.1 eV at the rim of the waveguide and exhibits an evanescent decay with a decay length of 120 nm (Figure 2(d)). The emitted photon energy perfectly matches the phase-matching criterion explained by equation (1) and demonstrated in Figure 1(b). Within the helical arm, the CL intensity also shows an evanescent tail, decreasing to a minimum at a distance of 185 nm from the rim and increasing again towards the opposite rim. At this opposing rim, located at a position 1.2 μm from the origin, the intensity is higher, creating a double-peak feature. This double-peak behavior is attributed to a slight misalignment of the helix, causing it to deviate from being perfectly parallel to the beam trajectory (Figure S1). The maximum CL intensities observed at the waveguide rims confirm the efficient coupling of the electron beam to the plasmonic mode.

The CL intensity remains at 60 % of its maximum amplitude at the helix center, where the electron beam does not interact directly with the material and is approximately 1.3 μm away from the waveguide rim. The asymmetrical coupling to the plasmonic modes at the opposing rims, combined with the higher CL intensity observed when the electron travels through the axial void, indicates a significant coupling of the evanescent tails of the plasmonic fields within the axial void. The resulting modal structure facilitates efficient electron-photon interactions, even in regions where the electron is 1 μm away from the rim of the waveguide.

The observed radiation from the helix thus originates from the coupling of the moving electron to a plasmonic mode supported by the gold-coated helical waveguide, rather than to photonic modes confined within the low-index fiber core. In a straight fiber configuration, this coupling leads to guided-mode excitation without far-field emission. However, the helical geometry introduces periodic variations in the interaction distance, enabling phase-matched emission via a combined Smith–Purcell and Cherenkov-like mechanism. The curvature of the helix, with a bending radius approximately three times the effective wavelength of the plasmonic mode, facilitates partial leakage of the guided energy into free space along the waveguide, resulting in directional far-field radiation.

**Figure 3:**
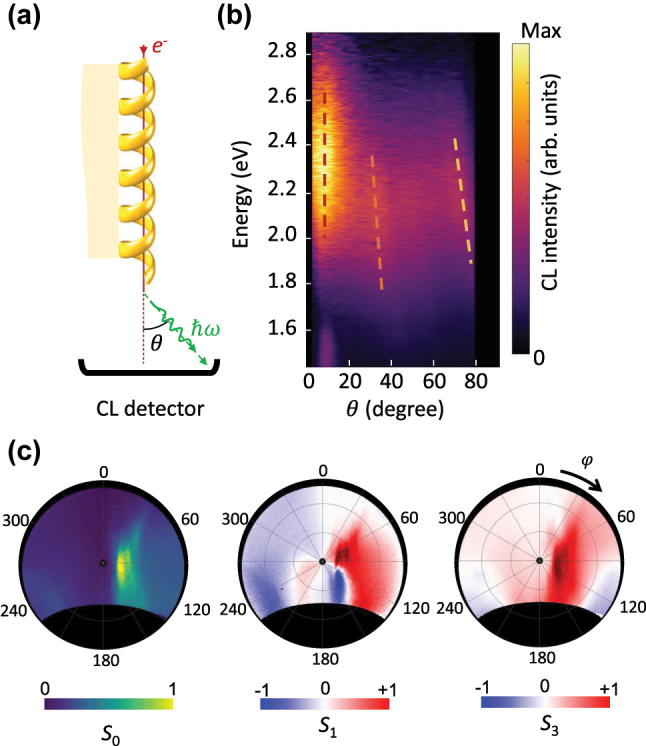
Directional and elliptically polarised radiation for phase-matched interaction between the electron beam and the helical waveguide. (a) The schematic of the experiment. (b) Angle-resolved CL spectra, and (c) angle-resolved Stokes parameters of the radiation measured at the integrated photon energy range of 2.15 eV–2.26 eV. Electron beam has the kinetic energy of 18 keV and propagates parallel to the helix axis at the impact position of *r*_*h*_ + *b* + 20 nm, forming a spot size of 100 nm.

To further illustrate the angular range of the emission, the CL emission is resolved versus the polar angle of the emission and photon energy, by performing momentum-resolved spectroscopy (Figure 3(a) and (b)) [37]. The azimuthal angle is set to the direction of strongest emission – i.e., *φ* = ±10°, as confirmed by the angle-resolved emission map presented later in Figure 3(c).

The angle-resolved spectral CL map displays a broad peak at the energy of 2.1 eV–2.5 eV, collimated along the symmetry axis of the grating. The dark regions at *θ* = 0 and at *θ* > 80° correspond to the hole implemented into the mirror for avoiding electron beam contamination and damage, and the restricted collection efficiency of the mirror due to the its limited size, respectively. This collimated emission and its photon energy agrees well with the *m* = 0 emission (dashed red line in Figure 1(b)). The higher-order diffraction orders are obvious as well with faint directional emissions at higher angles, marked by dashed orange (*m* = 1) and dashed yellow (*m* = 2) lines in Figure 3(b).

The excitation of the helix with the electron beam parallel to the helix axis leads to a chiral polarization following the helicity of the structure. The emission profile therefore follows as well the handedness of the helix, in such a way that the emission is predominantly in the form of right-handed circularly polarized light, where the detector convention is considered. Measured stokes parameters, namely $S_{0}={\left|A_{x}\right|}^{2}+{\left|A_{y}\right|}^{2}$ (light intensity), $S_{1}={\left|A_{x}\right|}^{2}-{\left|A_{y}\right|}^{2}$ , and S3=2ImAx*Ay verify the generation of directional and right-handed circularly-polarized light (Figure 3(c)). In the above equations, *A*_*x*_ and *A*_*y*_ depict the complex amplitudes of the *x*- and *y*-components of the electric field. For an elliptically-polarized light, where a phase offset exists between *A*_*x*_ and *A*_*y*_ components, *S*_3_ takes a non-zero value and its sign is associated with the handedness of the polarization (positive for right-handed and negative for left-handed polarization, when the detector convention is used).

**Figure 4:**
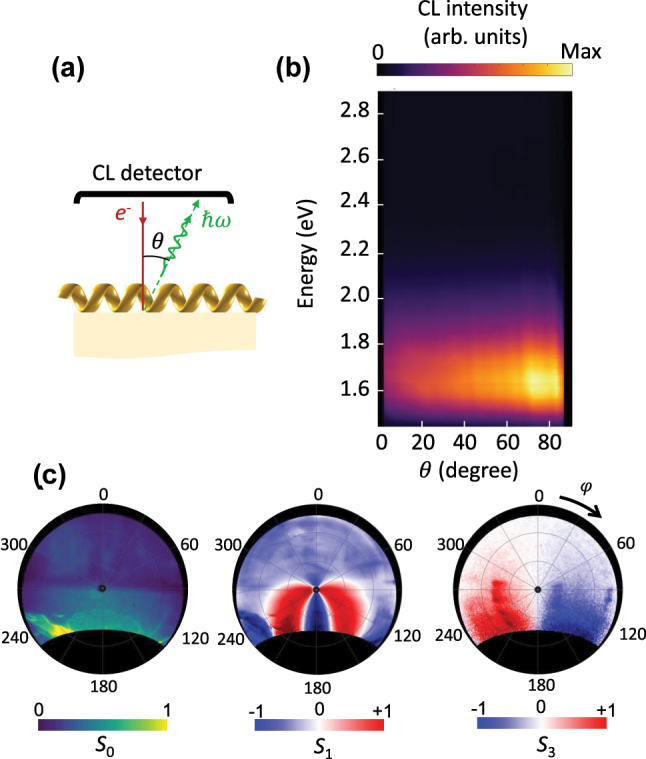
The radiation pattern and the polarisation states of light for an electron beam propagating perpendicular to the helical waveguide. (a) Schematic of the experiment. (b) Angle-resolved CL spectra and (c) angle-resolved Stokes parameters of the radiation measured at the integrated photon energy range of 1.6 eV–1.7 eV. The electron beam has the kinetic energy of 18 keV and propagates perpendicular to the helix axis.

To further demonstrate that the radiation originates from the combined Cherenkov and Smith–Purcell emission mechanism described by Eq. (1), we have performed angle-resolved spectroscopy and polarimetry measurements for the case where the electron beam traverses the helix perpendicular to its axis (Figure 4). In this configuration, the emission exhibits a distinct profile, peaking at 1.6 eV and extending toward higher angular ranges. The emitted photon energy in this case does not depend on the electron impact position. Since the helix is long, reflections from its ends are negligible, preventing the formation of standing-wave patterns within the helix. This indicates that the electron primarily couples to lower-energy modes of the waveguide, which emit from the waveguide’s ends, resulting in a broad angular distribution.

Polarimetry measurements further reveal significant differences in polarization between emissions from the two ends of the waveguide. The left end predominantly emits right-handed polarized light, while the right end emits left-handed polarized light. These results align with observations reported elsewhere and can be attributed to the dependence of the emission’s chirality on the electron beam’s impact position [38].

The emergence of circular polarization in the emitted light can be attributed primarily to the geometrical chirality of the helical waveguide. While the supported plasmonic mode possesses an azimuthal dependence (mode number *n* = 1; see Figure 1b), which reflects its orbital angular momentum, its polarization state is not directly determined by this modal property of the straight fiber. Instead, the helical geometry induces a rotating plasmonic surface current, which introduces a dynamic phase shift between the transverse components of the radiated field. This mechanism acts analogously to a quarter-wave plate, effectively generating circular polarization. Experimental observations and numerical simulations confirm that the handedness of the helix governs the handedness of the emitted circularly polarized light.

Noticeably, the emission from the helix in the case when electron travels parallel to the helix axis, cannot be described by pure Smith–Purcell radiation. There is a large mismatch between the momentum of the free-space light and the momentum of the current-density distribution of the moving electron at the kinetic energy of 18 keV. Therefore, to satisfy the Smith–Purcell criterion, namely, $\omega \;v_{e}^{-1}=k_{0}\cos\theta +2m\pi \Lambda^{-1}$ , diffraction orders of 13 ≤ *m* ≤ 35 is required, for the photon energy within 1 eV and 3 eV, which makes the interaction rather inefficient.

**Figure 5:**
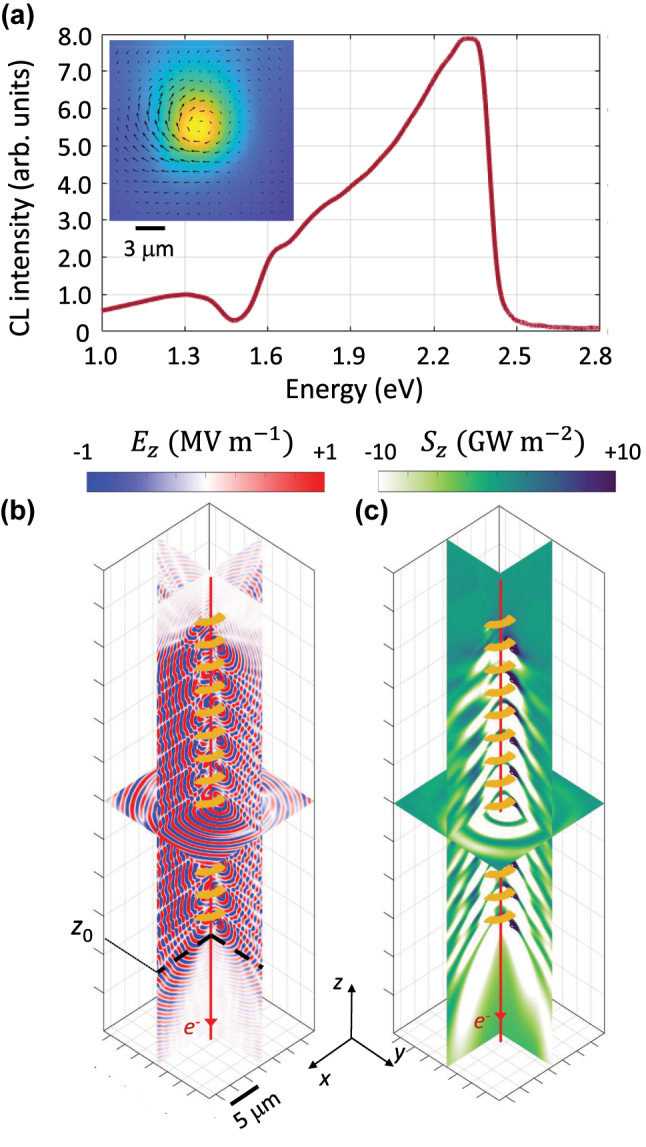
Simulation results, exhibiting the interaction of a moving electron at the kinetic energy of 18 eV interacting with the helix. The inset shows the spatial profile of the *z*-component of the electric field at *z* = *z*_0_. The inset displays the spatial profile of absolute value of the transverse electric field at the *xy*-plane marked by *z*_0_, positioned at 3 μm below the helix end. (a) Radiation spectrum, calculated at the *xy*-plane positioned at 8 μm below the helix. The spatial profile of the (b) *z*-component of the electric field and (c) the *z*-component of the Poynting vector, at *E* = 2.22 μm.

The simulations of a moving electron interacting with the helix reproduce the main observed features. These simulations were performed using a finite-difference time-domain (FDTD) solver developed in our group [39]. The large size of the structure, combined with its fine features (e.g., the gold layer thickness and confined plasmon polaritons), posed significant computational challenges. We discretised the entire volume using a 15 nm grid size and introduced 20,000 time iterations. A second-order radiation boundary condition was employed to eliminate the need for additional volume required by perfectly matched layers. The calculated spectrum of the light intensity (Poynting vector) at surface positioned 8 μm below the helix demonstrates a broad resonance centered at *E* = 2.2 eV in a good agreement with observed experimental results. The emission from the helix is also collimated downward, making an angle of approximately 20° with the electron beam trajectory. The transverse field intensity ( ${\left|E_{x}\right|}^{2}+{\left|E_{y}\right|}^{2}$ ) has a collimated spatial profile and constitutes an elliptical polarization (Inset of Figure 5(a)). The field profiles are symmetrical distributed with respect to the helix axis, in contrast with the experiments, due to the presence of the plateau in the latter that is required to hold the helix.

Similar to the Cherenkov radiation and the Smith–Purcell effect, the energy of the emitted photons can be tuned via the electron kinetic energy. Supplementary Note S3 and Figure S2 indicate that this indeed can be observed as a control parameter.

Based on comparisons between the experimental results and the phase-matching condition – where the phase constant of the optical modes is assumed to correspond to that of a straight optical fiber – we conclude that the optical modes are only slightly modified by transforming the waveguide into a helical structure. This is justified by the relatively large helical pitch of the helix compared to the fiber’s radius, which supports this assumption.

The performance of the helical waveguides – particularly emission efficiency and polarization characteristics – can be influenced by fabrication imperfections such as surface roughness or slight asymmetries. To evaluate the impact of these tolerances, we conducted cathodoluminescence measurements across multiple fabricated helices. The consistency observed in spectral profiles, angular distributions, and polarization states suggests that the emission characteristics are robust with respect to minor fabrication variations.

Finally, helical structures have previously been proposed to generate helically shaped optical wavefronts. However, our helical waveguide takes advantage of the guided modes of a 3D-printed waveguide to generate phase-matched radiation from electron beams in the visible range, unlike the generation of terahertz Smith–Purcell radiation [40], [41], [42].

## 3 Conclusions

In conclusion, we have established a new phase-matching scenario that enables stronger electron–photon interactions. This is achieved by maintaining synchronicity between the waveguiding modes of an optical fiber made into a helix and electron beams through the introduction of extended optical paths and multiple sequential interactions. The structure is fabricated using two-photon-polymerization-based 3D printers (Quantum X series, NanoScribe Company), enabling a new paradigm for strong electron–photon interactions. Our phase-matching scenario could be modified for even stronger interactions, e.g., by inserting the helix in a hollow cylindrical waveguide. In an inverse approach [43], [44], [45], launching optical waves into the fiber and leveraging laser-induced near-field interactions could enable regimes of ultra-strong electron–photon interactions, which are highly favorable for dielectric laser accelerators.

## Supplementary Material

Supplementary Material Details
