# Supplementary material for: Phase-matched electron–photon interactions enabled by 3D-printed helical waveguides
Source: Nanophotonics. 2025 Sep 10;14(20):3279–86. doi: 10.1515/nanoph-2025-0297 (PMC12588561; doi:10.1515/nanoph-2025-0297)
Supplement: Supplementary file 1 — Supplementary Material Details [file j_nanoph-2025-0297_suppl_001.pdf]

Supplementary Information for:

Phase-matched electron-photon interactions enabled with 3D-printed helical waveguides

Masoud Taleb,[1] Mohsen Samadi,[2] and Nahid Talebi[1,*]

[1]Institute of Experimental and Applied Physics, Kiel University, 24098 Kiel, Germany
[2]Department of Electrical and Information Engineering, Kiel University, 24143Kiel, Germany

Content:

1- Optical modes of the polymer/gold fiber
2- Images of the helical waveguide and sample holder
3- Dependence of the emission wavelength on the electron's kinetic energy

1- Optical modes of the polymer/gold fiber

To construct the optical modes of the system, we employ the vector potential approach in the cylindrical coordinate system, with ρ, φ, and z being the radius in *xy*-plane, azimuthal angle, and the *z*-axis respectively [1]. Optical modes in fibers exhibit a hybrid nature; that is, the modes are not purely transverse except when $n=0$, where *n* represents the azimuthal degree of freedom [2]. The fiber is composed of a polymer core with the permittivity $\varepsilon_{r1}=2.63$ within the region specified by $\rho<a$ and a gold thin cladding with the permittivity ε_{r2} [3] within the region $a<\rho<b$, whereas the region $\rho>b$ is considered to be vacuum with $\varepsilon_{r3}=1$. The solution Ansatz constitutes spatial distributions of the magnetic vector potential, as

$$A_z=\begin{cases} C_1 I_n(\kappa_1\rho)e^{in\varphi}e^{-ik_z z} & \rho<a \\ \left(C_2 I_n(\kappa_2\rho)+C_3 K_n(\kappa_2\rho)\right)e^{in\varphi}e^{-ik_z z} & a<\rho<b, \\ C_4 K_n(\kappa_3\rho)e^{in\varphi}e^{-ik_z z} & \rho>b \end{cases} \tag{S1}$$

and the electric vector potential, as

$$F_z=\begin{cases} D_1 I_n(\kappa_1\rho)e^{in\varphi}e^{-ik_z z} & \rho<a \\ \left(D_2 I_n(\kappa_2\rho)+D_3 K_n(\kappa_2\rho)\right)e^{in\varphi}e^{-ik_z z} & a<\rho<b, \\ D_4 K_n(\kappa_3\rho)e^{in\varphi}e^{-ik_z z} & \rho>b \end{cases} \tag{S2}$$

where C_i and D_i are unknown coefficients to be obtained via satisfying the boundary conditions. k_z is the propagation constant of the waves propagating along the z – axis, and the characteristic equations in all three regions are $-\kappa_i^2+k_z^2=\varepsilon_{ri}k_0^2$, with i = 1, 2, and 3. I_n and K_n are the modified Bessel functions of the first and second kinds with order n.

The electric and magnetic field coefficients are obtained as

$$\vec{E}(\vec{r}) = -\vec{\nabla}\times\vec{F} + \left(i\omega\varepsilon_0\varepsilon_r\right)^{-1}\left(\vec{\nabla}\times\vec{\nabla}\times\vec{A}\right) \tag{S3}$$

and

$$\vec{H}(\vec{r}) = +\vec{\nabla}\times\vec{A} + \left(i\omega\mu_0\right)^{-1}\left(\vec{\nabla}\times\vec{\nabla}\times\vec{F}\right), \tag{S4}$$

Respectively, with both $\vec{A}$ and $\vec{F}$ vectors composed of only z-components. After obtaining the field coefficients by using equations (S1) to (S2), the tangential boundary conditions are satisfied, and therefore the following 4 coupled equations are obtained that relates C_2, C_3, D_2, and D_3 as

$$\begin{aligned}
&+\left[\kappa_2^2-\kappa_1^2\right]\frac{1}{i\omega\varepsilon_0\varepsilon_{r2}}\frac{nk_z}{a}I_n(\kappa_2 a)I_n(\kappa_1 a)C_2+\left[\kappa_2^2-\kappa_1^2\right]\frac{1}{i\omega\varepsilon_0\varepsilon_{r2}}\frac{nk_z}{a}K_n(\kappa_2 a)I_n(\kappa_1 a)C_3\\
&+\kappa_1\kappa_2\left\{\kappa_2 I_n^{'}(\kappa_1 a)I_n(\kappa_2 a)-\kappa_1 I_n^{'}(\kappa_2 a)I_n(\kappa_1 a)\right\}D_2\\
&+\kappa_1\kappa_2\left\{\kappa_2 I_n^{'}(\kappa_1 a)K_n(\kappa_2 a)-\kappa_1 K_n^{'}(\kappa_2 a)I_n(\kappa_1 a)\right\}D_3=0,
\end{aligned} \tag{S5}$$

$$\begin{aligned}
&+\left[\kappa_2^2-\kappa_3^2\right]\frac{1}{i\omega\varepsilon_0\varepsilon_{r2}}\frac{nk_z}{b}I_n(\kappa_2 b)K_n(\kappa_3 b)C_2+\left[\kappa_2^2-\kappa_3^2\right]\frac{1}{i\omega\varepsilon_0\varepsilon_{r2}}\frac{nk_z}{b}K_n(\kappa_2 b)K_n(\kappa_3 b)C_3\\
&+\kappa_2\kappa_3\left[\kappa_2 K_n^{'}(\kappa_3 b)I_n(\kappa_2 b)-\kappa_3 I_n^{'}(\kappa_2 b)K_n(\kappa_3 b)\right]D_2\\
&+\kappa_2\kappa_3\left[\kappa_2 K_n^{'}(\kappa_3 b)K_n(\kappa_2 b)-\kappa_3 K_n^{'}(\kappa_2 b)K_n(\kappa_3 b)\right]D_3=0,
\end{aligned} \tag{S6}$$

$$\begin{aligned}
&\kappa_1\kappa_2\left[-\frac{\varepsilon_{r1}}{\varepsilon_{r2}}\kappa_2 I_n^{'}(\kappa_1 a)I_n(\kappa_2 a)+\kappa_1 I_n^{'}(\kappa_2 a)I_n(\kappa_1 a)\right]C_2\\
&+\kappa_1\kappa_2\left[-\frac{\varepsilon_{r1}}{\varepsilon_{r2}}\kappa_2 I_n^{'}(\kappa_1 a)K_n(\kappa_2 a)+\kappa_1 K_n^{'}(\kappa_2 a)I_n(\kappa_1 a)\right]C_3\\
&+\left[\kappa_2^2-\kappa_1^2\right]\frac{1}{i\omega\mu_0}\frac{nk_z}{a}I_n(\kappa_2 a)I_n(\kappa_1 a)D_2+\left[\kappa_2^2-\kappa_1^2\right]\frac{1}{i\omega\mu_0}\frac{nk_z}{a}K_n(\kappa_2 a)I_n(\kappa_1 a)D_3=0,
\end{aligned} \tag{S7}$$

and

$$\begin{aligned}
&\kappa_2\kappa_3\left[-\frac{\varepsilon_{r3}}{\varepsilon_{r2}}\kappa_2 K_n^{'}(\kappa_3 b)I_n(\kappa_2 b)+\kappa_3 I_n^{'}(\kappa_2 b)K_n(\kappa_3 b)\right]C_2\\
&+\kappa_2\kappa_3\left[-\frac{\varepsilon_{r3}}{\varepsilon_{r2}}\kappa_2 K_n^{'}(\kappa_3 b)K_n(\kappa_2 b)+\kappa_3 K_n^{'}(\kappa_2 b)K_n(\kappa_3 b)\right]C_3\\
&+\left[\kappa_2^2-\kappa_3^2\right]\frac{1}{i\omega\mu_0}\frac{nk_z}{b}I_n(\kappa_2 b)K_n(\kappa_3 b)D_2+\left[\kappa_2^2-\kappa_3^2\right]\frac{1}{i\omega\mu_0}\frac{nk_z}{b}K_n(\kappa_2 b)K_n(\kappa_3 b)D_3=0.
\end{aligned} \tag{S8}$$

Equations (S5) to (S8) defines a nonlinear Eigenvalue problem, for obtaining the propagation constant k_z and the eigenvectors C_2, C_3, D_2, and D_3 .The unknown coefficients C_1, C_4, D_1, and D_4 are obtained as

$$C_1 = +\frac{\varepsilon_{r1}}{\varepsilon_{r2}}\frac{\kappa_2^2}{\kappa_1^2}\frac{I_n(\kappa_2 a)}{I_n(\kappa_1 a)}C_2+\frac{\varepsilon_{r1}}{\varepsilon_{r2}}\frac{\kappa_2^2}{\kappa_1^2}\frac{K_n(\kappa_2 a)}{I_n(\kappa_1 a)}C_3, \tag{S9}$$

$$C_4 = +\frac{\varepsilon_{r3}}{\varepsilon_{r2}} \frac{\kappa_2^2}{\kappa_3^2} \frac{I_n(\kappa_2 b)}{K_n(\kappa_3 b)} C_2 + \frac{\varepsilon_{r3}}{\varepsilon_{r2}} \frac{\kappa_2^2}{\kappa_3^2} \frac{K_n(\kappa_2 b)}{K_n(\kappa_3 b)} C_3, \tag{S10}$$

$$D_1 = +D_2 \frac{\kappa_2^2}{\kappa_1^2} \frac{I_n(\kappa_2 a)}{I_n(\kappa_1 a)} + D_3 \frac{\kappa_2^2}{\kappa_1^2} \frac{K_n(\kappa_2 a)}{I_n(\kappa_1 a)}, \tag{S11}$$

and

$$D_4 = +D_2 \frac{\kappa_2^2}{\kappa_3^2} \frac{I_n(\kappa_2 b)}{K_n(\kappa_3 b)} + D_3 \frac{\kappa_2^2}{\kappa_3^2} \frac{K_n(\kappa_2 b)}{K_n(\kappa_3 b)},$$

Which are used to calculate the spatial distribution of the electric and magnetic fields in all regions.

2. Images of the helical waveguide and sample holder

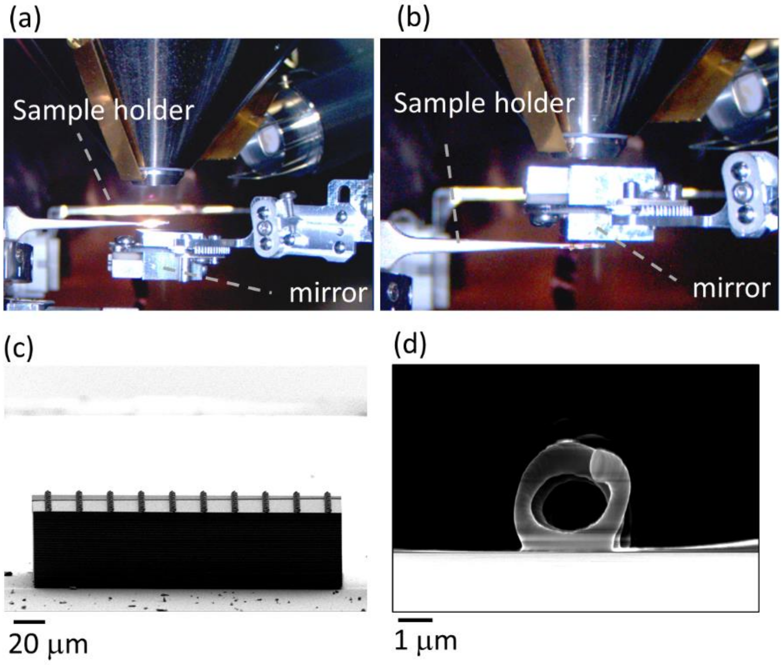

FIG. S1. (a) The setup, consisting of a sample holder inside a scanning electron microscope and a parabolic mirror positioned below the sample. (b) The same setup with the parabolic mirror positioned above the sample. (c) A scanning electron (SE) image of the sample, showing a series of microhelices arranged on top of a plateau. (d) A high-magnification top-view image of a single helix, demonstrating the alignment of the helix axis parallel to the electron trajectory.

3. Dependence of the emission wavelength on the electron's kinetic energy

The phase-matching condition defined in Eq. (1) of the main text enables control over the photon energy and intensity for a specific helix through various parameters, including the electron's group velocity and the diffraction order *m*. Fig. S2 illustrates that the emitted photon energy is indeed dependent on the electron's kinetic energy. For an electron with the kinetic energy of 15 keV ($v_e = 0.24c$, where c is the light speed in vacuum), only a faint emission is observed, which is two orders of magnitude weaker than the emission from a 17 keV electron beam. For the latter, the emission occurs at the peak photon energy of E = 2.2 eV, in a good agreement with the phase-matching condition. For an electron beam with the kinetic energy of 20 keV ($v_e = 0.27c$), the peak photon energy occurs at E = 2.4 eV, whereas the phase-matching condition specifies an emission at the energy of 2.65 eV. The emission angle is higher than that observed for an electron with a kinetic energy of 17 keV. Both observations indicate that the emission corresponds to the m = 1 diffraction order. This condition results in an emission at a photon energy of 2.5 eV, which aligns better with the experimental observations.

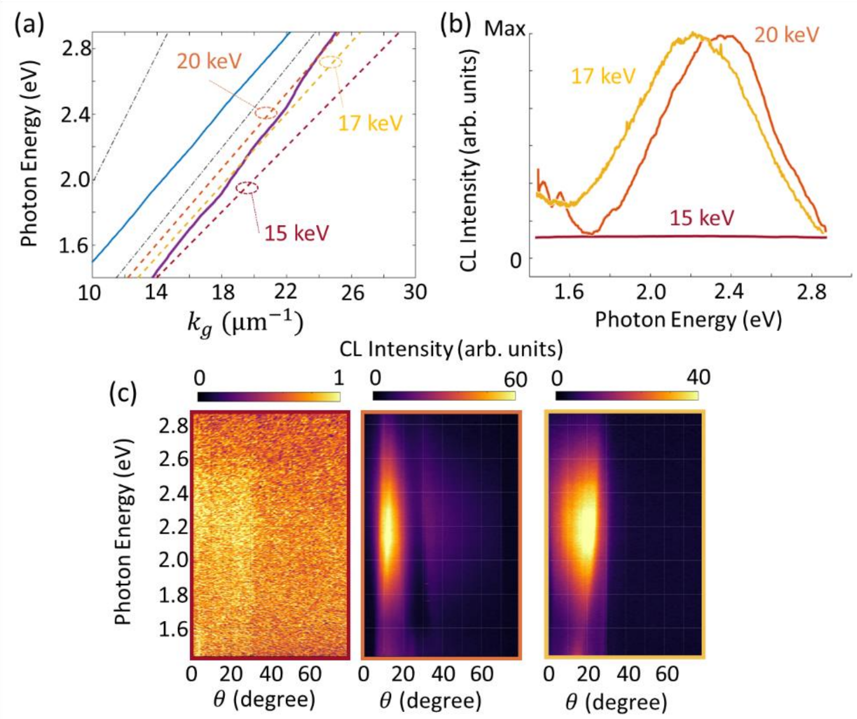

FIG. 3. (a) Dispersion diagram of the fundamental mode (Solid purple line) and the second mode (solid blue line) of the optical fiber. Dashed-dotted lines display the optical lines in vacuum and in the polymer. The colored dashed lines exhibit the phase-matching condition for an electron with depicted kinetic energies propagating parallel to the helix axis. (b) CL spectra and (b) CL angle-resolved spectral maps for an electron with the kinetic energies of 15 keV (left), 17 keV (middle), and 20 keV (right) propagating parallel to the helix.

References:

[1] R. F. Harrington, *Time-harmonic electromagnetic fields* (mcGraw-Hill Book Company, New York, 1961).

[2] A. H. Cherin, *Introduction to Optical Fibers* (McGraw-Hill, US, 1982).

[3] P. B. Johnson and R. W. Christy, Physical Review B **6**, 4370 (1972).
